# Antibacterial and Antiyeast Compounds from Marine-Derived Bacteria

**DOI:** 10.3390/md12052913

**Published:** 2014-05-13

**Authors:** Muhammad Abdul Mojid Mondol, Hee Jae Shin

**Affiliations:** 1 Institute for Organic and Biomolecular Chemistry, Georg-August University, Tammannstrasse 2, Göttingen D 37077, Germany; E-Mail: mondol_sst@yahoo.com; 2 Marine Natural Products Chemistry Laboratory, Korea Institute of Ocean Science & Technology, 787 Haeanro, Ansan 426-744, Korea; 3 Department of Marine Biotechnology, Korea University of Science and Technology, 217 Gajungro, Daejeon, 305-350, Korea

**Keywords:** antibiotics, marine microorganisms, NMR data, antimicrobial activity

## Abstract

Two new (**2** and **3**) and a known (**1**) antimicrobial compounds were isolated from EtOAc extracts of two marine bacterial strains cultured in modified Bennett’s broth medium. The structures of these compounds were determined based on the analysis of nuclear magnetic resonance (NMR), high resolution mass spectroscopy (HRMS), literature data review and considering biogenesis. All the compounds (**1**–**3**) demonstrated *in vitro* antimicrobial activities against selected pathogenic strains.

## 1. Introduction

In spite of remarkable progress in medicine, infectious diseases caused by bacteria, fungi and viruses are a major threat to human health. Poor people, especially from developing countries who are exposed to unhygienic conditions in their daily activities, are the worst sufferers of infectious diseases. Their sufferings have been increased many-fold due to prolonged illness caused by widespread drug resistant pathogens (e.g., multidrug-resistant *Staphylococcus aureus*, vancomycin resistant enterococcal strains with 73% mortality rate) and cost of treatment [1,2,3]. It has been estimated that more than 70% of pathogenic bacteria are resistant to at least one existing antibiotic [4]. As a result of a steady increase in drug-resistant pathogens every year, there have been demands for the development of new and effective antimicrobial drugs.

Most of the currently used natural product-derived therapeutics has been originated from terrestrial sources. However, mining marine diversified samples will clearly help for the discovery of novel drugs as well. A comparative analysis showed that natural products obtained from marine sources are chemically more diversified compare to the terrestrial natural products [5]. As a result, scientists have expanded their research from land to ocean (70% of the Earth’s surface) in order to find new natural leads for drug candidates [6].

**Figure 1:**
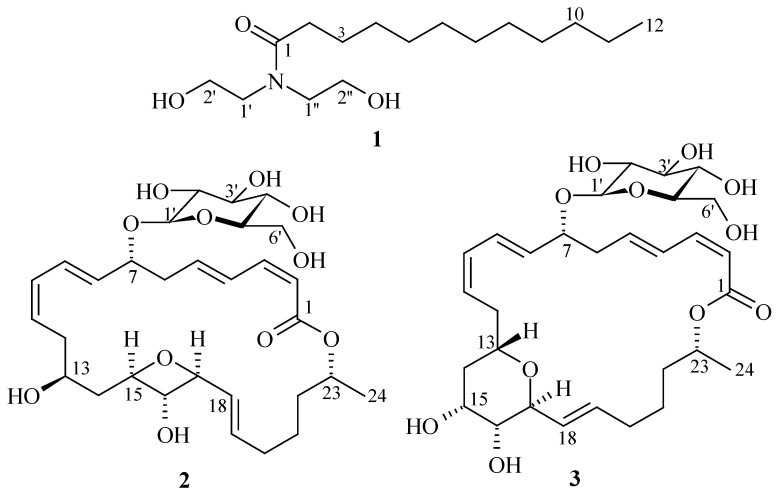
Structures of antimicrobial compounds (**1**–**3**).

A number of biologically active compounds (anticancer, antimicrobial, antifouling, *etc.*) have been isolated from marine sources [7]. Some of these bioactive molecules have already been selected for treating various diseases and many of them are under clinical investigations [6,8]. Marine microorganisms, living in challenging environments and competing with each other for space and nutrition, are potential sources for the discovery of new bioactive metabolites. As a part of our ongoing research program, we isolated three antimicrobial compounds (**1**–**3**) from marine bacteria (Figure 1). Here, we report the isolation, structure determination and antimicrobial activities of **1**–**3**.

## 2. Results and Discussion

**Figure 2:**
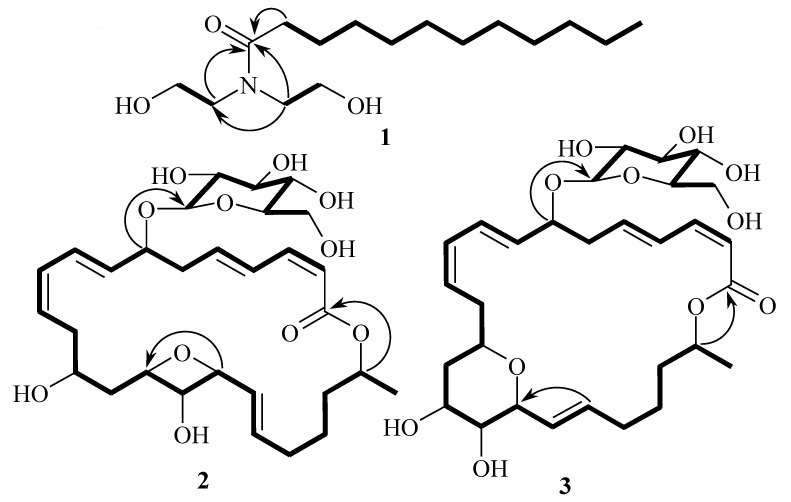
COSY (bold lines) and key HMBC (arrows) correlations for **1**–**3**.

Compound **1** has been reported as a synthetic compound [9]. However, few spectroscopic data were available to positively confirm the structure of **1** from the original synthetic work. Since this is the first report of **1** from natural sources, the structure determination and spectroscopic data are reported herein. Compound **1** was obtained as an amorphous solid which gave a quasimolecular ion [M + Na]^+^ at *m*/*z* 310.2349 in the positive mode HRESIMS, consistent with the molecular formula C_16_H_33_NO_3_. The terminal UV absorption at 202 nm suggested that **1** had an amide carbonyl group. The IR absorption bands at 1043 and 1619 cm^−1^ indicated the presence of carbonyl functionality and C–N bond, respectively. By analysis of the COSY spectrum, three ^1^H spin systems were identified, from H_2_-2 to H_2_-12, from H_2_-1′ to H_2_-2′, and from H_2_-1″ to H_2_-2″ (Figure 2).

The methylene proton signals from H_2_-4 to H_2_-11 were overlapped. An 1′,1″-azanediyldiethanol moiety was established by COSY correlations between H_2_-1′ and H_2_-2′, and between H_2_-1″ and H_2_-2″, and an HMBC correlation of H_2_-1′′ with C-1′ (Figure 2). The 1′,1″-azanediyldiethanol moiety was attached to the carbonyl carbon C-1 (δ_C_ 176.8) as H_2_-1′ and H_2_-1″ showed HMBC correlations with C-1 (Figure 2). The structure of **1** was established as *N*,*N*-bis(2-hydroxyethyl)-dodecanamide and known as lauramide diethanolamine.

Glycosylated macrolactins A1 (**2**) and B1 (**3**) were isolated as amorphous solids by repeated chromatographic steps. The molecular formula of both compounds was determined to be C_30_H_44_O_11_ based on HRESIMS (*m*/*z* 603.2775 [M + Na]^+^) (**2**) and (*m*/*z* 603.2776 [M + Na]^+^) (**3**) in combination with ^1^H and ^13^C NMR data having nine degrees of unsaturation and compounds **2** and **3** were isomers of each other.

Detailed interpretation of all spectroscopic data (UV, IR, ID and 2D NMR) indicated that the structure of macrolactin A1 (**2**) was almost identical with 15,17-epoxy-16-hydroxy macrolactin A and macrolactin B1 (**3**) was with that of 13,17-epoxy-16-hydroxy macrolactin A [10] except for the down-field shift of H-7 (from δ_H_ 4.25 in 15,17-epoxy-16-hydroxy macrolactin A to δ_H_ 4.51 in **2** and from δ_H_ 4.29 in 13,17-epoxy-16-hydroxy macrolactin A to δ_H_ 4.54 in **3**) and additional signals for sugar moiety in ^1^H and ^13^C NMR spectra of both compounds (Table 1 and Supplementary Information). The sugar moiety in both **2** and **3** was attached to C-7 as indicated by an HMBC correlation between an anomeric proton H-1′ and C-7 (Figure 2).

**Table 1:** ^1^H and ^13^C NMR data of compounds **1**–**3**.

No.	1 ^a^		2 ^a^		3 ^a^	
	δ_C_	δ_H_, mult. (*J* in Hz)	δ_C_	δ_H_, mult. (*J* in Hz)	δ_C_	δ_H_, mult. (*J* in Hz)
1	176.8		168.3		168.1	
2	34.3	2.43, t (7.5)	118.1	5.50, d (11.5)	118.1	5.51, d (11.5)
3	26.7	1.60, m	144.6	6.65, t (11.5)	144.6	6.30, t (11.5)
4–11/4	23.8–33.2	1.32–1.29, m	130.6	7.17, dd (15.5, 11.5)	130.6 ^b^	7.17, dd (15.0, 11.5)
12/5	14.5	0.89, t (7.0)	141.6	6.35, dt (15.5, 7.0)	141.3	6.27, dt (15.0, 7.5)
1′/6	50.3	3.49, t (6.0)	41.6	2.46, m; 2.57, m	41.5	2.54, m
2′/7	61.0	3.67, t (5.5)	78.3	4.51, m	77.9 ^b^	4.54, m
1″/8	52.7	3.54, t (6.0)	132.8	5.52, dd (15.5, 8.5)	135.6	5.60, dd (15.1, 8.0)
2″/9	60.9	3.69, t (5.5)	131.0	6.63, dd (15.5, 11.5)	129.8	6.67, dd (15.1, 11.0)
10			131.9	6.07, t (11.5)	130.6 ^b^	6.06, t (11.0)
11			130.6	5.46, dt (11.5, 5.0)	130.3	5.52, dt (11.0, 6.5)
12			35.4	2.60, m	34.3	2.06, m; 2.93, m
13			75.8	3.45, m	72.1	3.72, m
14			41.1	1.38, q (11.5); 1.95, m	35.7	1.77, m; 2.06, m
15			73.9	3.52, ddd (11.4, 9.0, 5.0)	67.7	3.86, m
16			77.6	2.90, dd (9.0, 9.0)	72.0	3.54, dd (4.3, 4.3)
17			80.0	3.42, dd (9.0, 5.0)	76.6	4.28, dd (4.3, 4.3)
18			129.0	5.64 ^b^ [5.62, dd (15.3, 5.0)] ^c^	128.4	5.42 dd (15.6, 4.3)
19			132.0	5.64 b [5.46, dt (15.3, 8.5)] c	135.6	5.65, dt (15.6, 8.0)
20			34.6	2.01, m; 2.07, m	33.7	2.06, m; 2.14, m
21			26.9	1.44, m; 1.52, m	26.0	1.48, m; 1.57, m
22			37.0	1.64, m	36.5	1.60, m; 1.67, m
23			73.0	4.93, m	72.4	4.97, m
24			20.0	1.25, d (6.5)	20.1	1.25, d (6.0)
1′			100.6	4.29, d (8.0)	101.2	4.31, d (8.0)
2′			75.1	3.22, dd (9.0, 8.0)	75.1	3.20, dd (8.7, 8.0)
3′			71.8	3.23, dd (9.0, 8.4)	71.7	3.27, dd (8.7, 8.5)
4′			78.1	3.32, dd (9.5, 8.4)	78.1	3.32, dd (9.0, 8.5)
5′			78.0	3.18, m	77.9 ^b^	3.18, m
6′			62.8	3.64, dd (12.0, 6.0) 3.85, dd (12.0, 2.0)	62.7	3.65, dd (12.0, 5.5) 3.86, dd (12.0, 2.0)

^a^ Determined in CD_3_OD; ^b^ Overlapping singals; ^c^ Determined in DMSO-*d*_6_.

The relative configurations of all disubstituted double bonds in **2** and **3** were same as those of 15,17-epoxy-16-hydroxy macrolactin A and 13,17-epoxy-16-hydroxy macrolactin A, respectively, based on coupling constants analyses (Table 1) [10]. The large coupling constants of the anomeric proton (Table 1) indicated β-glycosidic linkages. The protons attached to glucopyranosyl moiety were diaxially arranged as indicated by large coupling constants (Table 1). The glucopyranosyl moiety of both compounds was confirmed to have d-configuration as acid hydrolysates showed same *R*_f_ value with an authentic sample in same solvent system. Data were not taken to determine the absolute configurations of **2** and **3** due to limited amount of yield. It should be noted that glycosylated macrolactins A1 and B1, 15,17-epoxy-16-hydroxy macrolactin A, and 13,17-epoxy-16-hydroxy macrolactin A were isolated from the same strain. As all the macrolactins are produced by the same biosynthetic pathways [11] and the chemical shifts of stereocenters in macrolactone rings of compounds **2** and **3** were almost identical to their respective derivatives [10], they may have same absolute configurations like their analogs.

Surfactants and macrolactins are well known for their antimicrobial activities. Surfactant molecules exhibited antimicrobial activity by increasing porosity in the cytoplasmic membrane [12], whereas macrolactins exhibited antimicrobial activity by inhibiting peptidyl transferase [13]. In the case of surfactants, antimicrobial activities depend on the chain length (10–16 carbon atoms) of the lipophilic and polarity of hydrophilic (COOH < CHO < OH) groups. The lower chain length containing surfactants are more active against Gram-(–) bacteria and yeasts, whereas Gram-(+) bacteria are more affected by the longer chain surfactants [14]. Compound **1** belongs to small surfactant molecules.

Macrolactins are polyene cyclic macrolactones; most of them are produced by *Bacillus* sp. [15]. Macrolactins showed a wide range of biological activities such as antimicrobials, antiviral and anticancer [15]. The position of hydroxyl group (OH) or introduction of keto group (C=O) in the macrolactone ring affected antimicrobial activity of macrolactins [16]. The introduction of ester groups at C-7 showed strong antibacterial activity [17,18,19]. Compounds **2** and **3** were less active than their corresponding ether-containing macrolactins [10] (Table 2), probably due to the attachment of a sugar moiety at C-7 position of both compounds. However, sugar moiety containing macrolatins have more advantage over non-sugar containing macrolactins in terms of solubility in polar solvent.

**Table 2:** Minimum inhibitory concentrations (MICs) of **1**–**3**.

Test Organisms	MICs (μM/mL)				
	1	2	3	AZ	AmpB
*Bacillus* *subtilis*	0.055	0.027	0.055	0.005	-
*Escherichia coli*	0.055	0.220	0.220	0.005	-
*Pseudomonas aeruginosa*	0.110	0.055	0.055	0.005	-
*Staphylococcus aureus*	0.110	0.055	0.055	0.005	-
*Saccharomyces cerevisiae*	0.220	0.220	0.220	-	0.002

AZ: Azithromycin (positive control); AmpB: Amphotericin B (positive control); -: not tested.

Ceramides are essential components of the outermost layer of the epidermis, the stratum corneum, which acts as a physical barrier and helps keep the skin hydrated. The ceramides represent about 40% of the lipids in this layer. The main biological function of ceramides is to control how skin cells grow and differentiate [20]. Natural ceramides are very unstable substances that are costly to obtain, so synthetic ceramides (pseudoceramides) are frequently used instead. Pseudoceramides have been used to treat skin diseases such as atopic dermatitis, psoriasis, and glucocorticoid-induced epidermal atrophy [21]. Lauramide diethanolamine (**1**) has been used as a foaming agent in bath products like shampoos and hand soaps, and in cosmetics as an emulsifying agent. The antimicrobial activity of **1** has been reported here for the first time. Compound **1** had a similar structure to but was much smaller than a synthetic ceramide (sphingolipid E, SLE) [22], which was developed as skin care products by Kao, a Japanese cosmetic company. Although compound **1** showed moderate antimicrobial activities (Table 2) against selected pathogens, its chemical structure is unusual in natural sources and has a unique carbon skeleton different from existing antimicrobial agents. Compound **1** and/or its modified forms might be useful bioprobes for further development of effective pseudoceramides which will be helpful to treat skin diseases.

## 3. Experimental Section

### 3.1. General Experimental Procedures

General experiments were done according to previous report [10].

### 3.2. Isolation and Taxonomy of the Strain O6CH80 and 09ID194

The strains O6CH80 and 09ID194 were isolated from sediment samples collected from Chuuk, Federated States of Micronesia and Ieodo, Republic of Korea’s southern reef during expeditions in 2006 and 2009, respectively, according to the described procedure [10]. The strain 09ID194 was identified as *Bacillus* sp. (GenBank Accession No. JN048684) [10] whereas the strain 06CH80 as *Streptomyces* sp. (GenBank Accession No. KJ371985) based on their 16S rDNA sequence analyses.

### 3.3. Seed and Large-Scale Cultures of the Strains O6CH80 and 09ID194

The seed and large-scale cultures of the strain 09ID194 were performed according to the procedure mentioned earlier [10]. The seed and large-scale cultures of the strain O6CH80 were done similarly but only the differences were temperature (28 °C) and salinity (20 g/L). The strain 06CH80 was large-scale cultured (40 L) for 7 days and then harvested.

### 3.4. Extraction and Isolation

The production culture broth (40 L) of the strain 06CH80 was centrifuged and the supernatant was extracted with EtOAc (2 × 40 L). The EtOAc layer was concentrated to dryness using rotary evaporators at 40 °C. The residual suspension (2.6 g) was subjected to an ODS open column chromatography followed by stepwise gradient elution with MeOH-H_2_O (v/v) (1:4, 2:3, 3:2, 4:1 and 100:0) as eluent. The fractions eluted with MeOH-H_2_O (100:0 and 2:3 v/v) again subjected to ODS MPLC (medium pressure liquid chromatography) (75%–100% MeOH in H_2_O) to obtain nine subfractions (SF-1-9). By repeated HPLC steps, compound **1** was obtained in pure form (1.5 mg) from the subfraction SF-2. Extraction and isolation of compounds **2** and **3** were done according to the steps described in previous report [10]. The fraction eluted with EtOAc-MeOH (v/v) (4:1) was subjected to further fractionations by a semi-preparative silica HPLC (6% *n*-hexane in EtOAc with flow rate: 1.5 mL/min; detector: UV) to obtain thirteen subfractions (SF-1-13). Compounds **2** and **3** were purified on an analytical ODS HPLC from the subfraction SF-7 using an isocratic program: 60% MeOH in H_2_O (flow rate: 0.6 mL/min; detector: UV) to yield pure compounds **2** (1.0 mg) and **3** (1.1 mg), respectively.

Lauramide diethanolamine (**1**): Amorphous solid; UV (MeOH) λ_max_ (log ε) 202 (4.31) nm; IR (MeOH) ν_max_ 2942, 2857, 1619, 1043 cm^−1^; ^1^H and ^13^C NMR data (CD_3_OD), see Table 1; HRESIMS *m*/*z* 310.2349 [M + Na]^+^ (calcd for C_16_H_33_NO_3_Na, 310.2358).

Glycosylated macrolactin A1 (**2**): Amorphous solid; 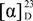 −97 (*c* 0.05, MeOH); UV (MeOH) λ_max_ (log ε) 237 (4.18) and 260 (4.15) nm; IR (MeOH) ν_max_ 3297 (br), 2941, 2830, 1600, 1022 cm^−1^; ^1^H and ^13^C NMR data (CD_3_OD), see Table 1; HRESIMS *m*/*z* 603.2775 [M + Na]^+^ (calcd for C_30_H_44_O_11_Na, 603.6535).

Glycosylated macrolactin B1 (**3**): Amorphous solid; 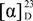 −88 (*c* 0.05, MeOH); UV (MeOH) λ_max_ (log ε) 236 (4.04) and 256 (4.20) nm; IR (MeOH) ν_max_ 3312 (br), 2941, 2830, 1600, 1022 cm^−1^; ^1^H and ^13^C NMR data (CD_3_OD), see Table 1; HRESIMS *m*/*z* 603.2776 [M + Na]^+^ (calcd for C_30_H_44_O_11_Na, 603.6535).

### 3.5. Acid Hydrolysis of Glycosylated Macrolactins 2 and 3

Compounds **2** and **3** (400 μg each) were refluxed in 1N HCl (1 mL) at 100 °C for 2 h separately. The completion of hydrolysis of **2** and **3** was confirmed by LC/MS analysis (in case of **2**, ESIMS, glycon *m*/*z* 179.03 [M − H]^−^ and aglycon *m*/*z* 417.15 [M − H]^−^; in case of **3**, ESIMS, glycon *m*/*z* 179.10 [M − H]^−^ and aglycon *m*/*z* 417.17 [M − H]^−^). After cooling, the reaction mixtures were extracted with EtOAc (2 × 2 mL). In both cases, the aqueous phases were neutralized with 1 N NaOH and evaporated to dryness. Glycons were analyzed by TLC (Kieselgel, eluting solvent CHCl_3_/MeOH 1:1, sprayed with 1% H_2_SO_4_ in vanillin and heated) to reveal the presence of d-glucose as their *R*_f_ values (0.55 in both cases) were coincident with the authentic sample. Optical rotation value of glycon obtained from **2** was 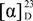 +41 (*c* 0.04, H_2_O) and from **3** was 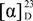 +44 (*c* 0.04, H_2_O).

### 3.6. Antimicrobial Activity Study

Antimicrobial activity study was performed according to the described method [10].

## 4. Conclusions

In conclusions, two microbial strains isolated from marine sediments were mass cultured in modified Bennet’s broth medium. Repeated chromatographic purifications of the EtOAc extracts obtained from the culture broths lead to the isolation of three antimicrobial compounds (**1**–**3**). All the compounds showed good antimicrobial activity. The structures of these compounds were uncommon and optimization of activities is required through derivatives synthesis with a view to develop antimicrobial agents.

## Supplementary Files

Supplementary File 1 Supplementary Information (PDF, 1433 KB)
