# Supplementary material for: Antibacterial and Antiyeast Compounds from Marine-Derived Bacteria
Source: Mar Drugs. 2014 May 13;12(5):2913–21. doi: 10.3390/md12052913 (PMC4052323; doi:10.3390/md12052913)
Supplement: Supplementary File 1 — Supplementary Information (PDF, 1433 KB) [file marinedrugs-12-02913-s001.pdf]

Supplementary Information

Figure S1. HRESIMS spectrum of compound **1**.

Elmt	Val.	Min	Max	Elmt	Val.	Min	Max	Use Adduct
H	1	0	300	O	2	0	12	Na
C	4	0	150					
N	3	0	12					

Error Margin (ppm):	10	DBE Range:	not fixed	Electron Ions:	both
HC Ratio:	unlimited	Apply N Rule:	yes	Use MSn Info:	no
Max Isotopes:	all	Isotope RI (%):	1.00	Isotope Res:	10000
MSn Iso RI (%):	75.00	MSn Logic Mode:	AND	Max Results:	10

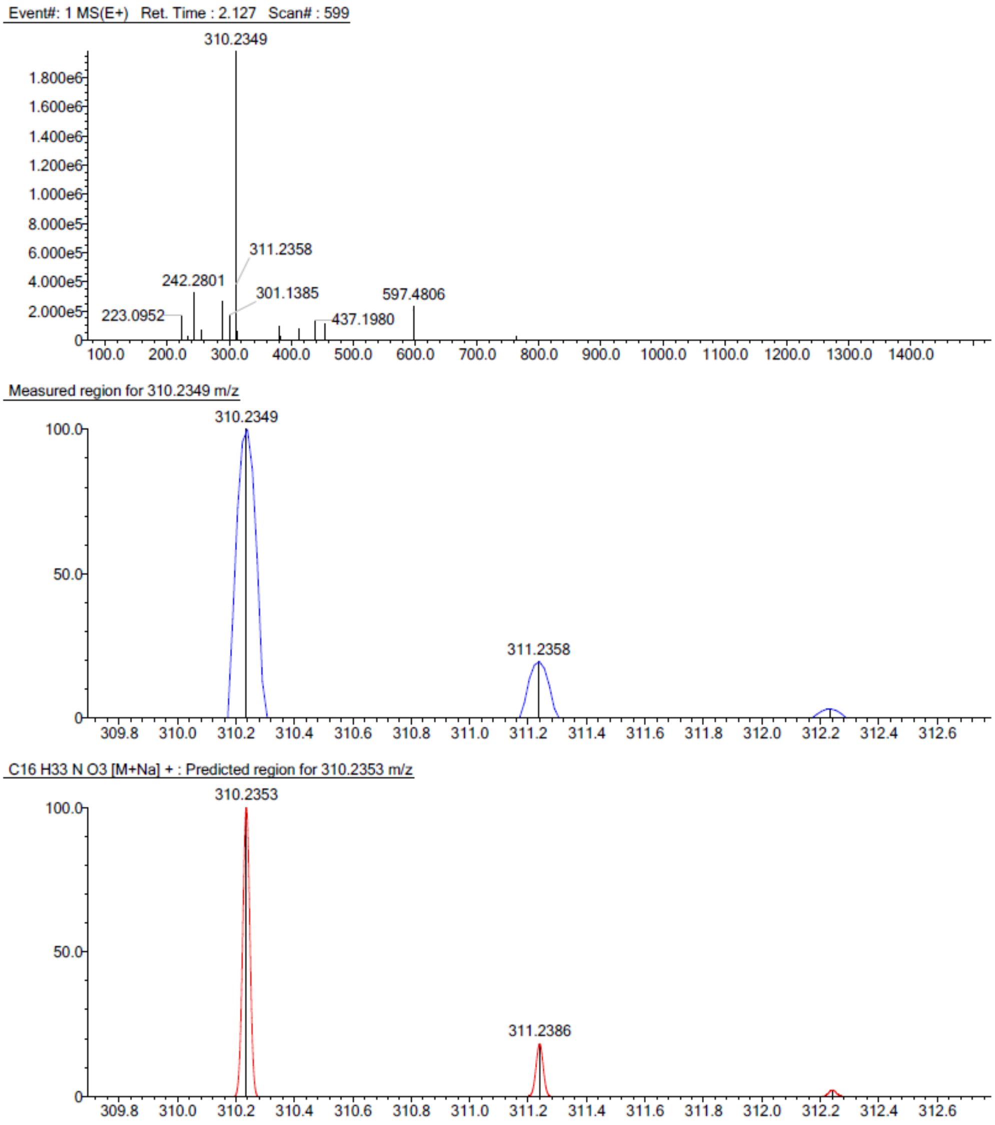

Rank	Score	Formula (M)	Ion	Meas. m/z	Pred. m/z	Df. (mDa)	Df. (ppm)	Iso	DBE
1	73.56	C16 H33 N O3	[M+Na] +	310.2349	310.2353	-0.4	-1.29	74.09	1.0

Figure S2. ^{1}H NMR spectrum of compound **1** recorded in CD_3OD at 500 MHz.

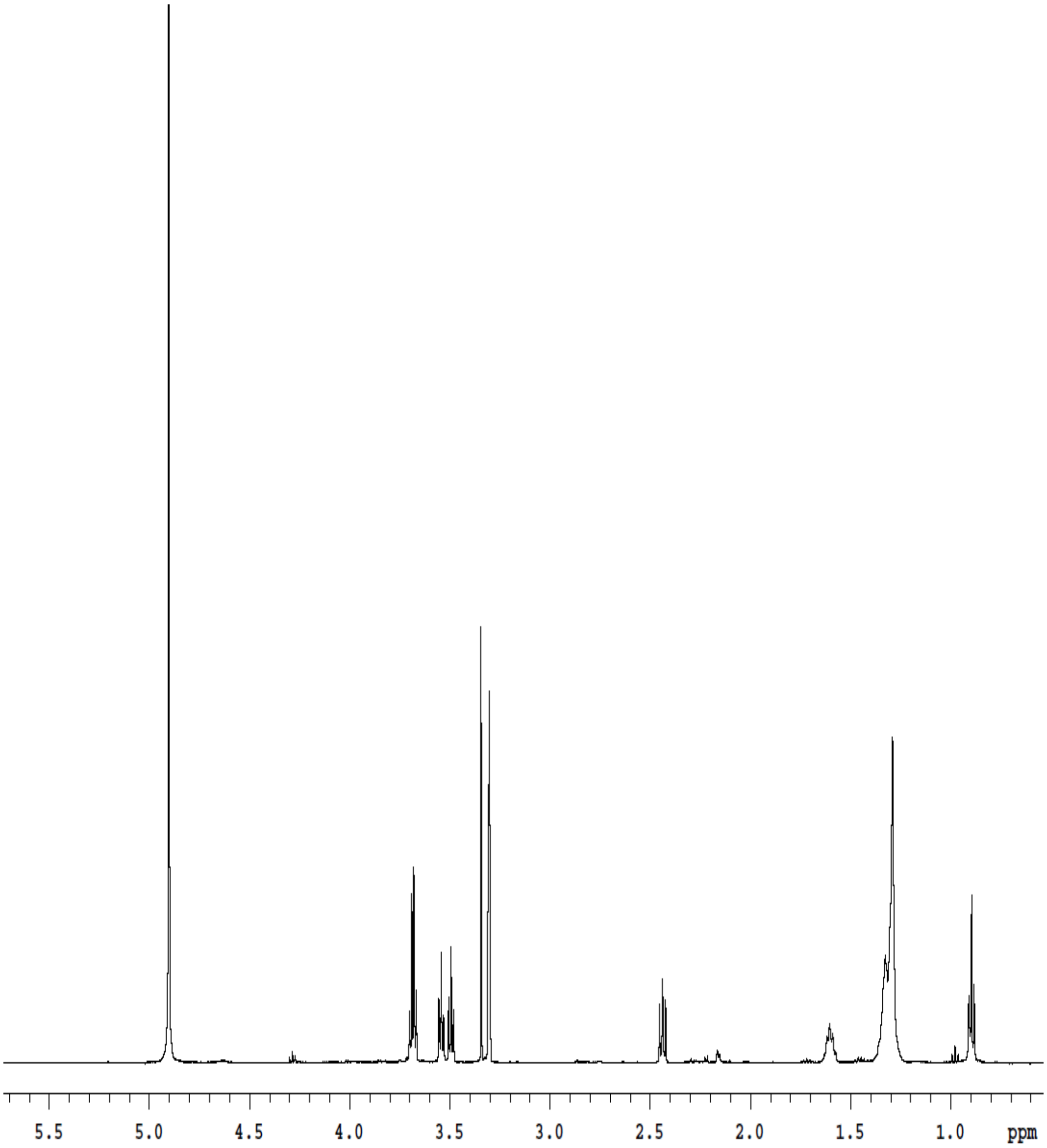

Figure S3. ^{13}C NMR spectrum of compound **1** recorded in CD_3OD at 125 MHz.

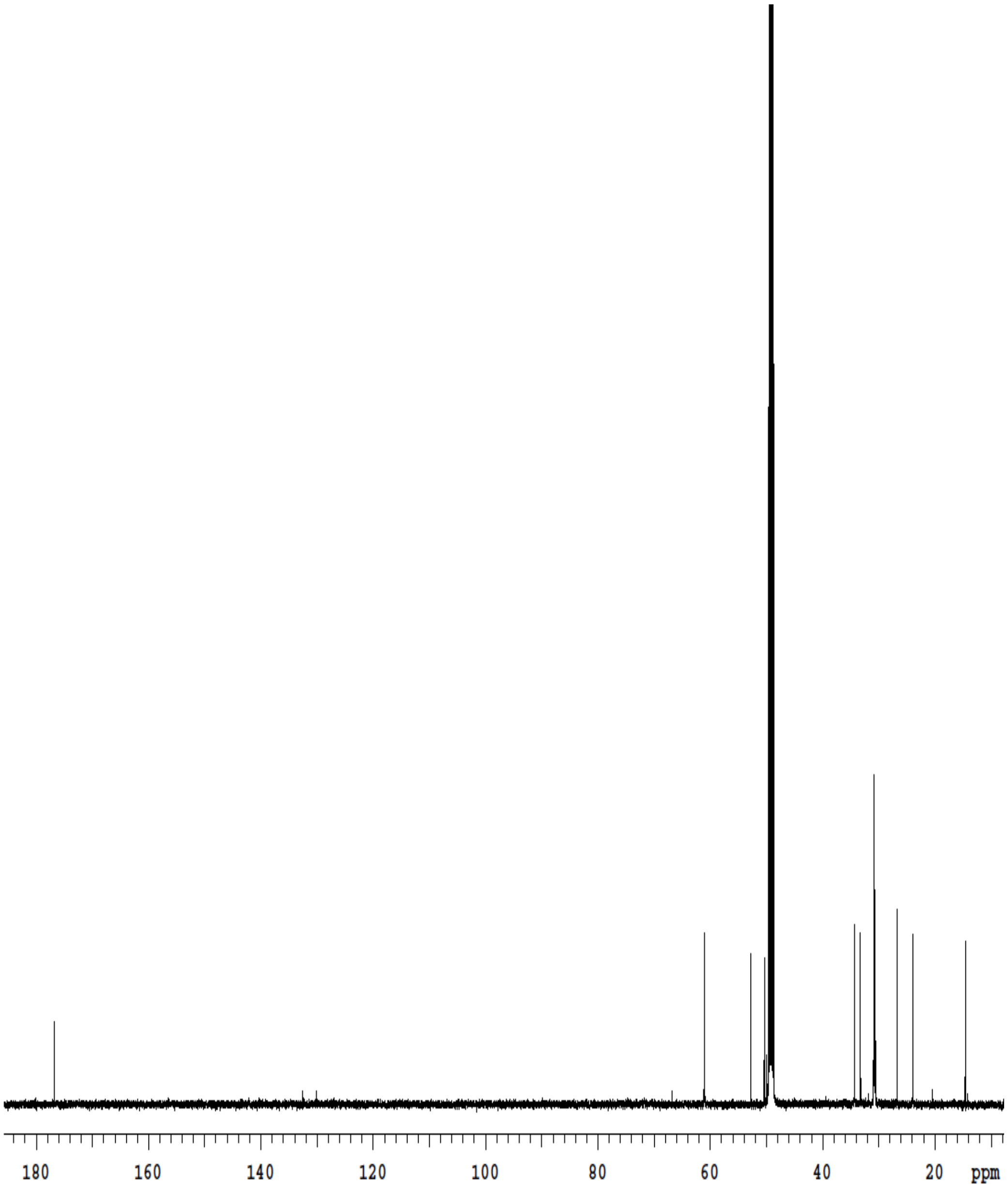

Figure S4. COSY spectrum of compound **1** in CD_3OD.

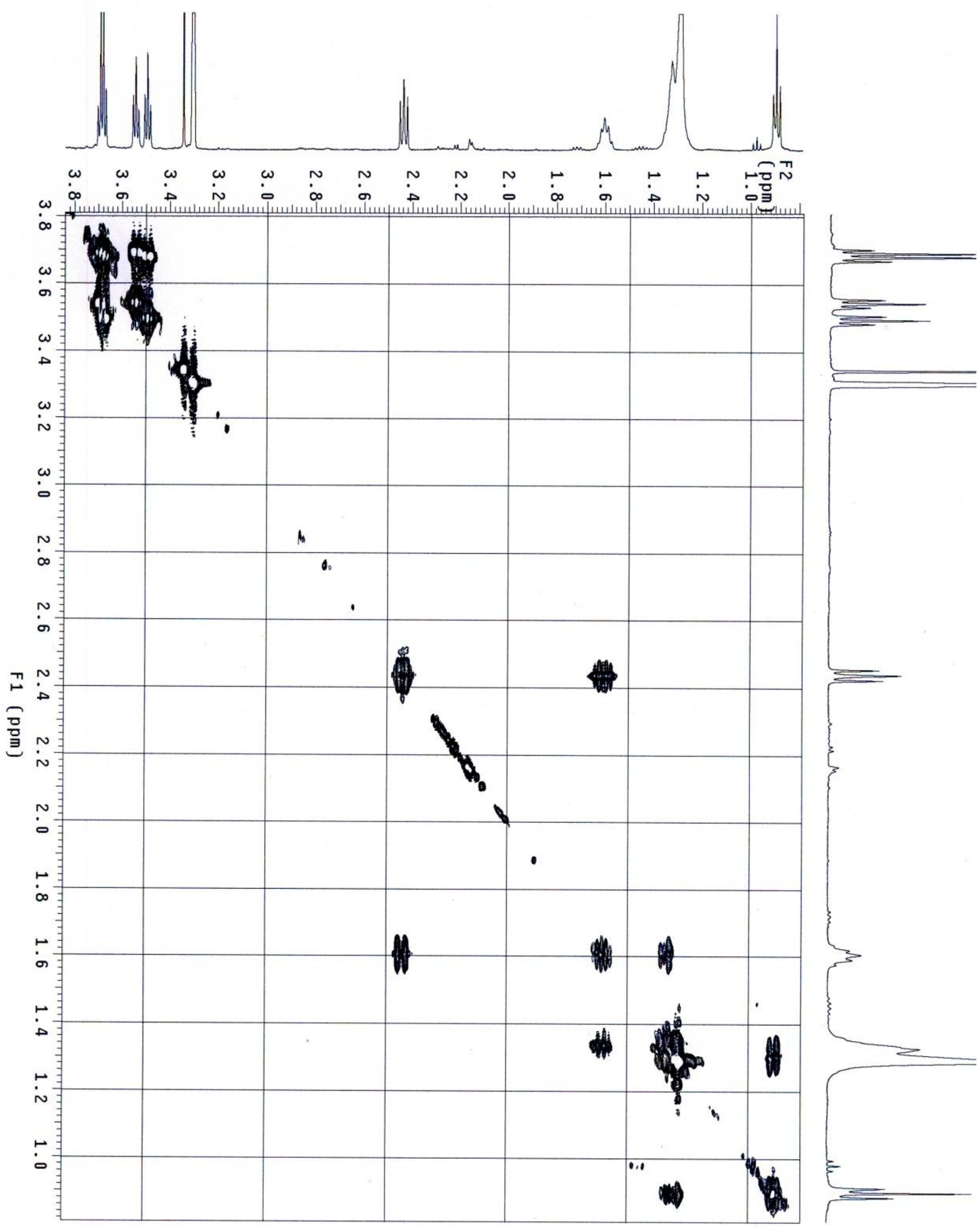

Figure S5. HSQC spectrum of compound **1** in CD_3OD.

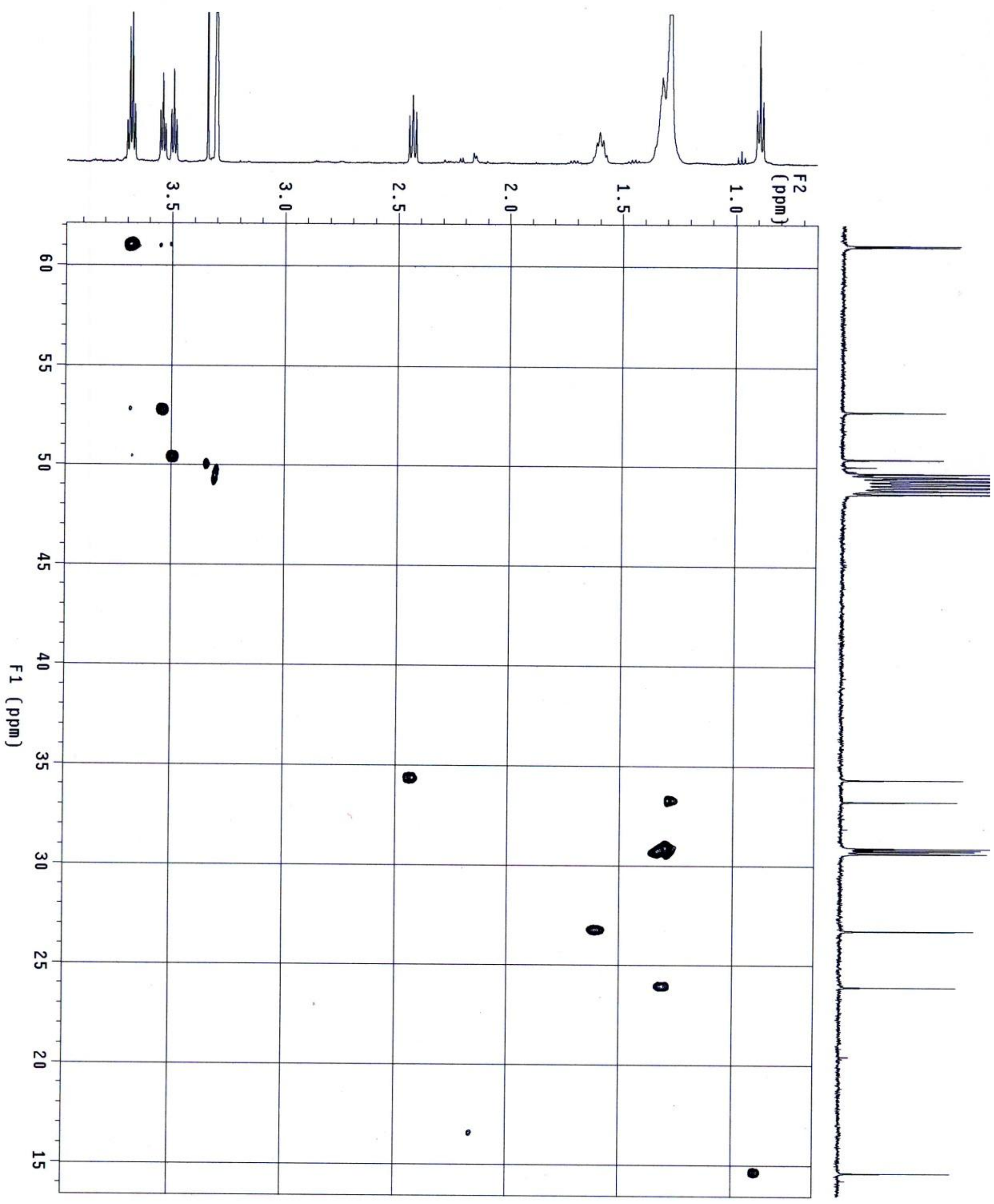

Figure S6. HMBC spectrum of compound **1** in CD_3OD.

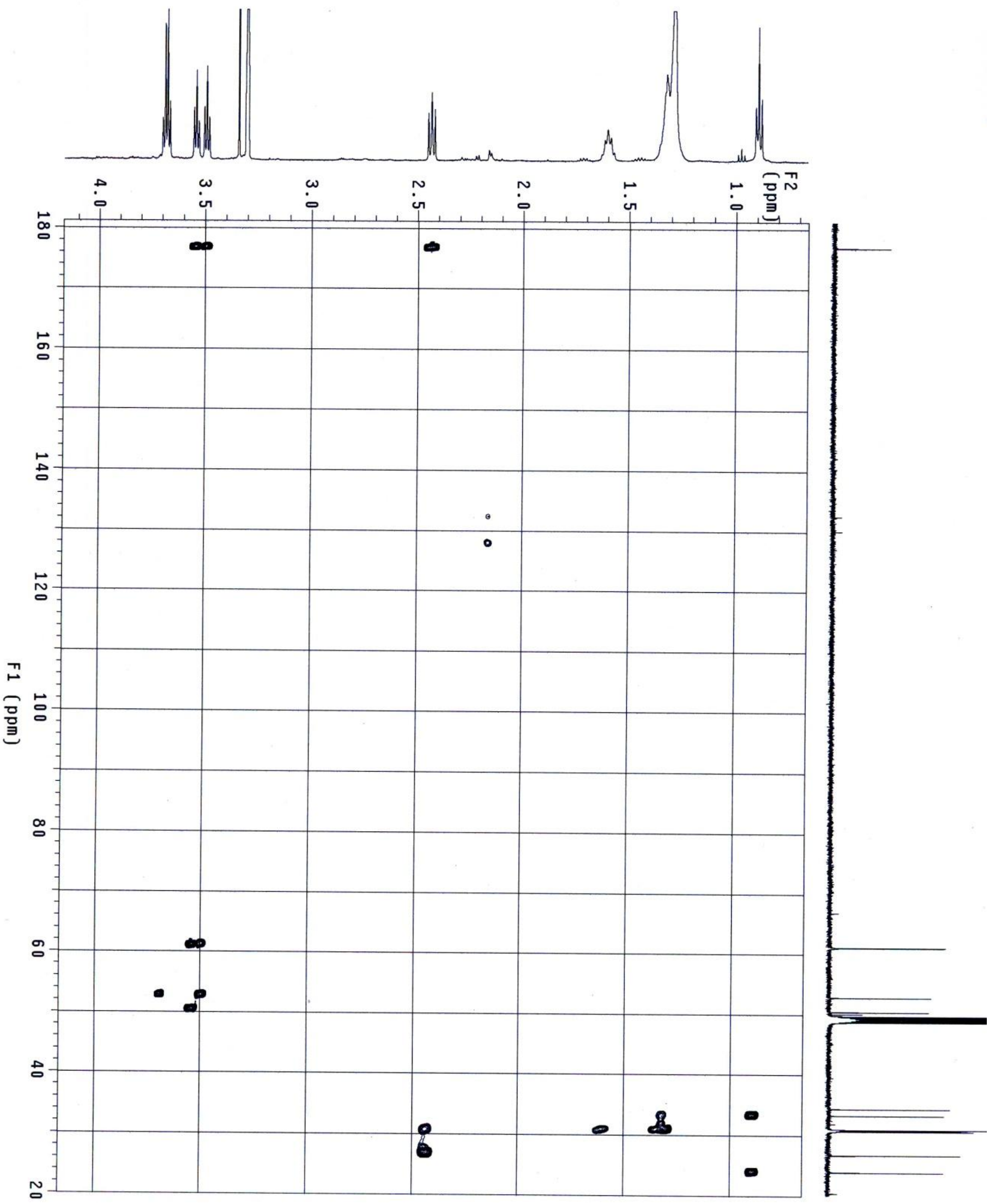

Figure S7. HRESIMS spectrum of compound **2**.

Elmt	Val.	Min	Max
H	1	0	300
C	4	0	150
N	3	0	0

Elmt	Val.	Min	Max	Use Adduct
O	2	0	12	Na

Error Margin (ppm):	10	DBE Range:	not fixed	Electron Ions:	both
HC Ratio:	unlimited	Apply N Rule:	yes	Use MSn Info:	no
Max Isotopes:	all	Isotope RI (%):	1.00	Isotope Res:	10000
MSn Iso RI (%):	75.00	MSn Logic Mode:	AND	Max Results:	10

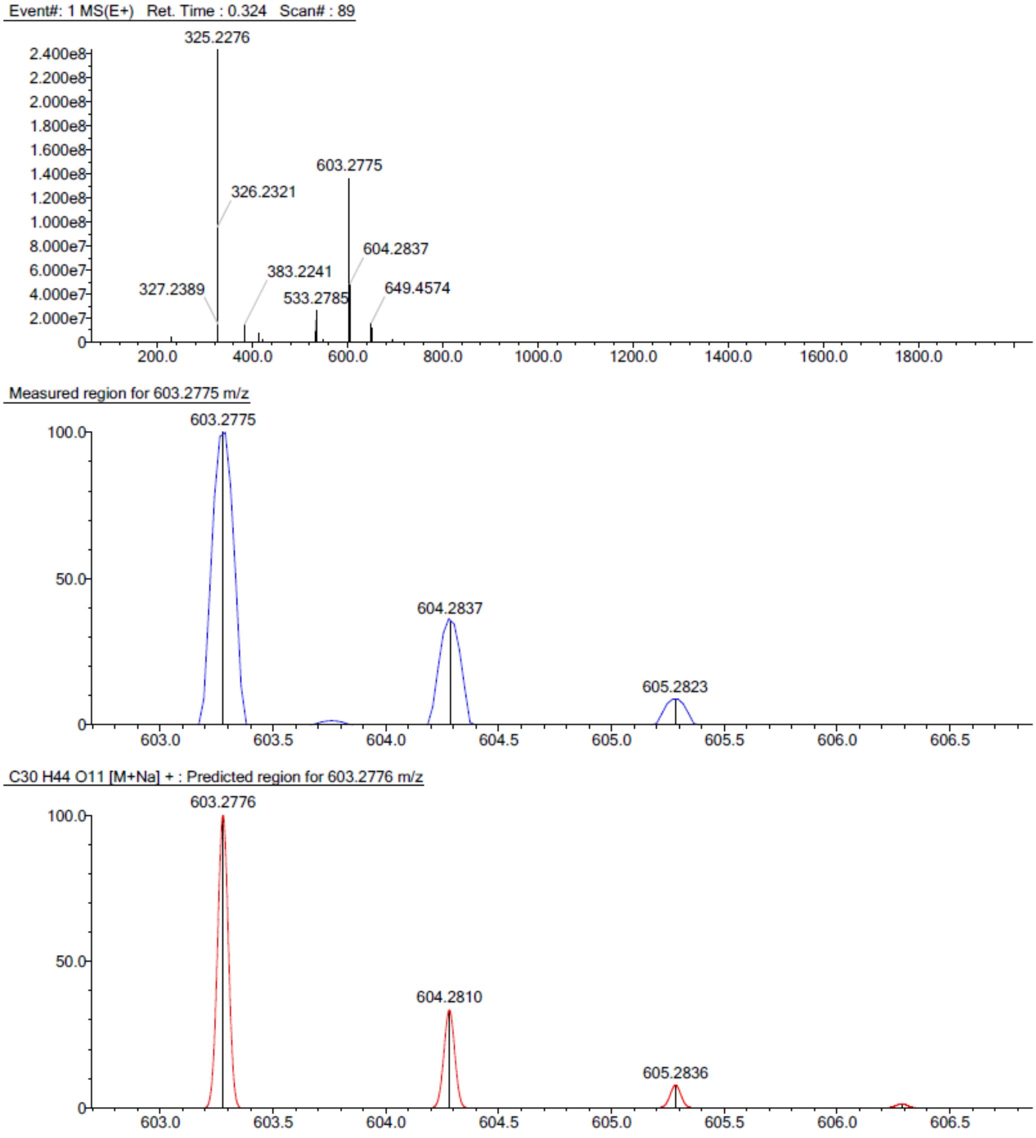

Rank	Score	Formula (M)	Ion	Meas. m/z	Pred. m/z	Df. (mDa)	Df. (ppm)	Iso	DBE
1	97.70	C30 H44 O11	[M+Na] +	603.2775	603.2776	-0.1	-0.17	97.70	9.0

Figure S8. ^{1}H NMR spectrum of compound **2** recorded in CD_3OD at 500 MHz.

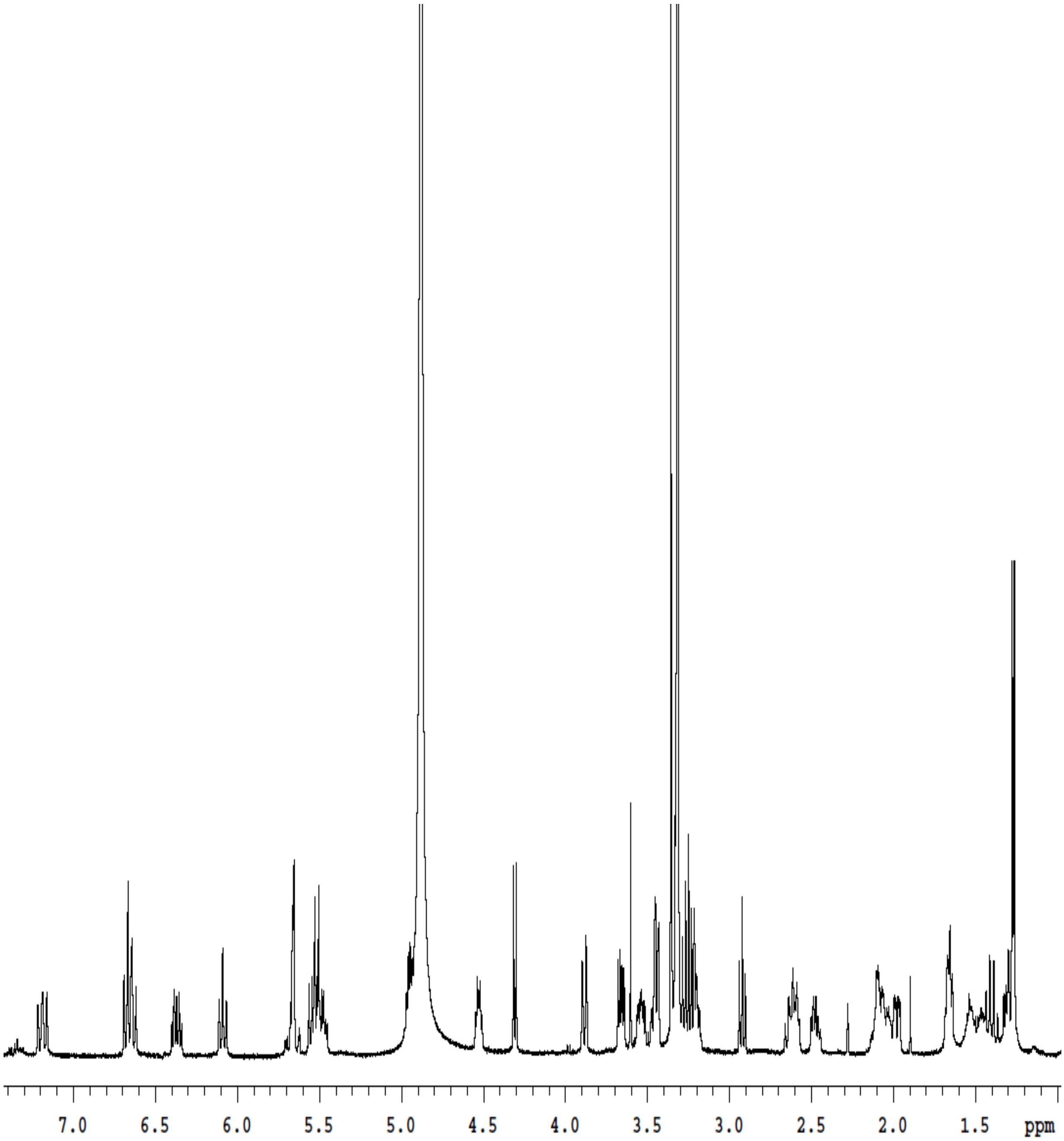

Figure S9. ^{13}C NMR of compound **2** recorded in CD_3OD at 125 MHz.

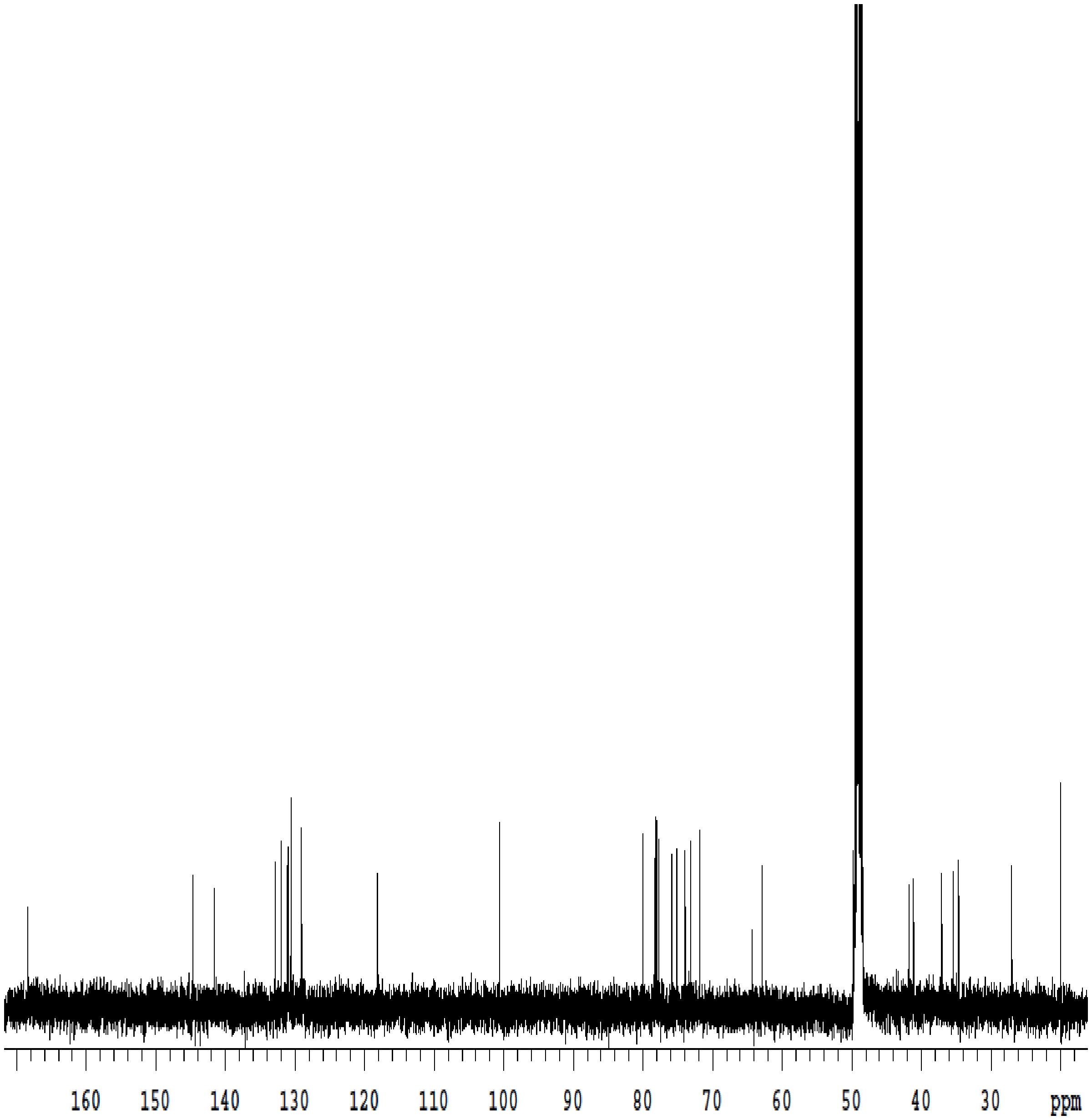

Figure S10. COSY spectrum of compound **2** in CD_3OD.

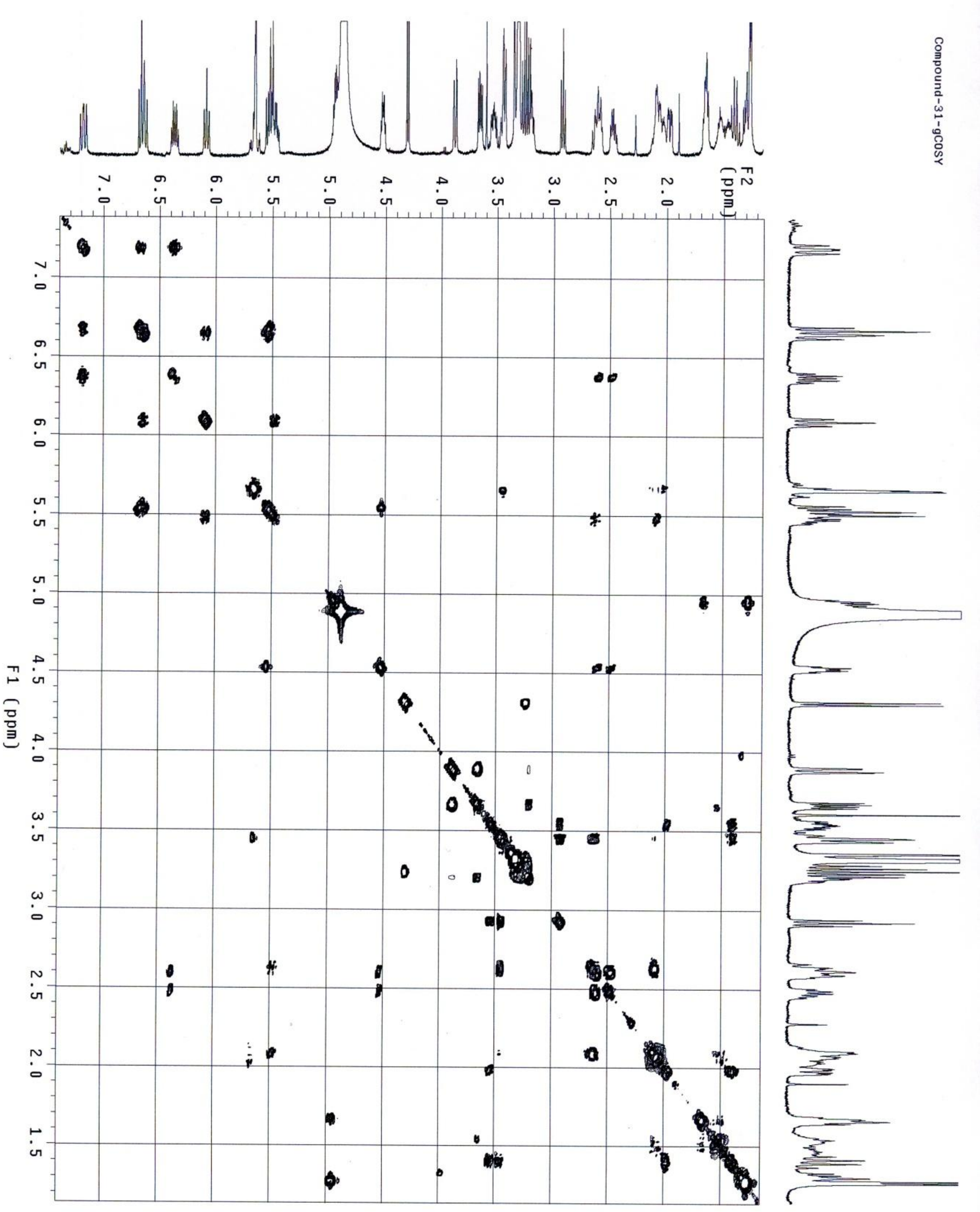

Figure S11. HSQC spectrum of compound **2** in CD_3OD.

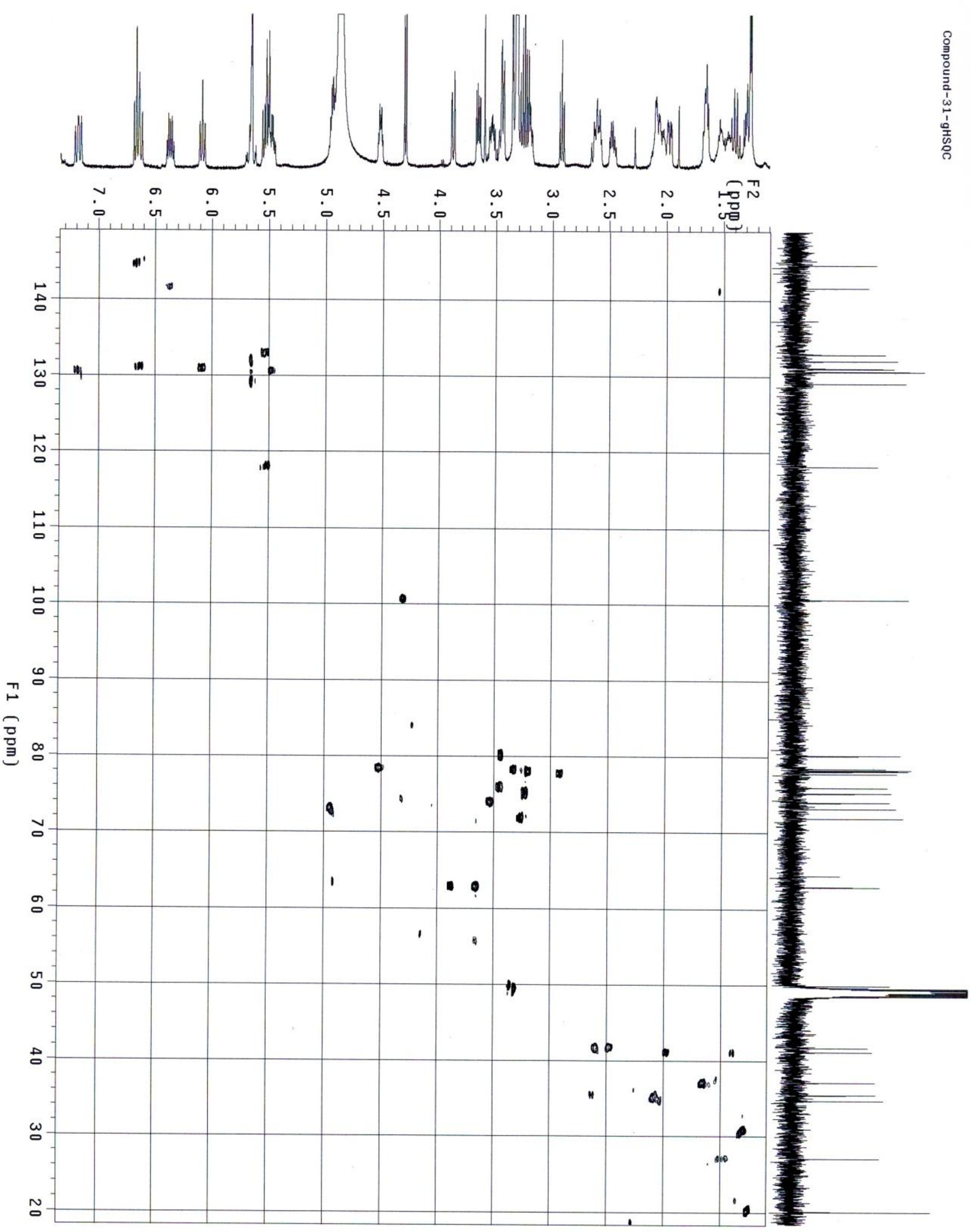

Figure S12. HRESIMS spectrum of compound **3**.

Elmt	Val.	Min	Max
H	1	0	300
C	4	0	150
N	3	0	0

Elmt	Val.	Min	Max
O	2	0	30

Use Adduct
Na

Error Margin (ppm):	10	DBE Range:	not fixed	Electron Ions:	both
HC Ratio:	unlimited	Apply N Rule:	yes	Use MSn Info:	no
Max Isotopes:	all	Isotope RI (%):	1.00	Isotope Res:	10000
MSn Iso RI (%):	75.00	MSn Logic Mode:	AND	Max Results:	10

Event#: 1 MS(E+) Ret. Time : 1.920 Scan# : 547

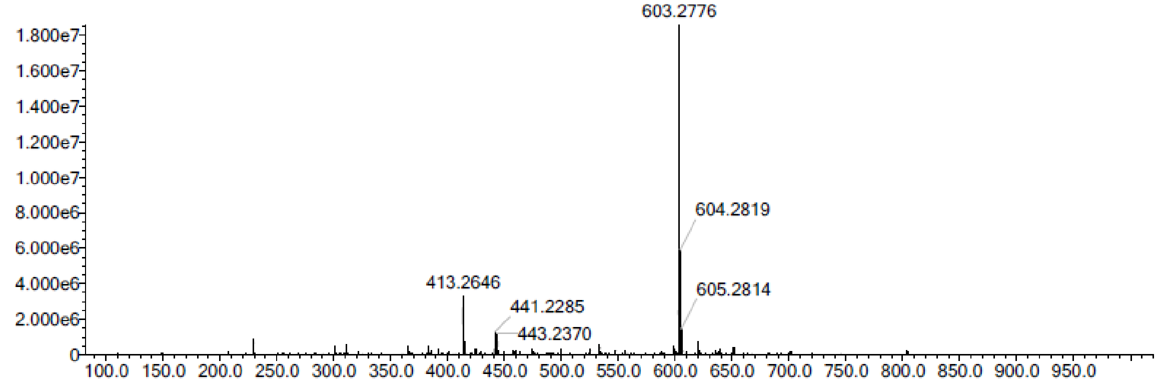

Measured region for 603.2776 m/z

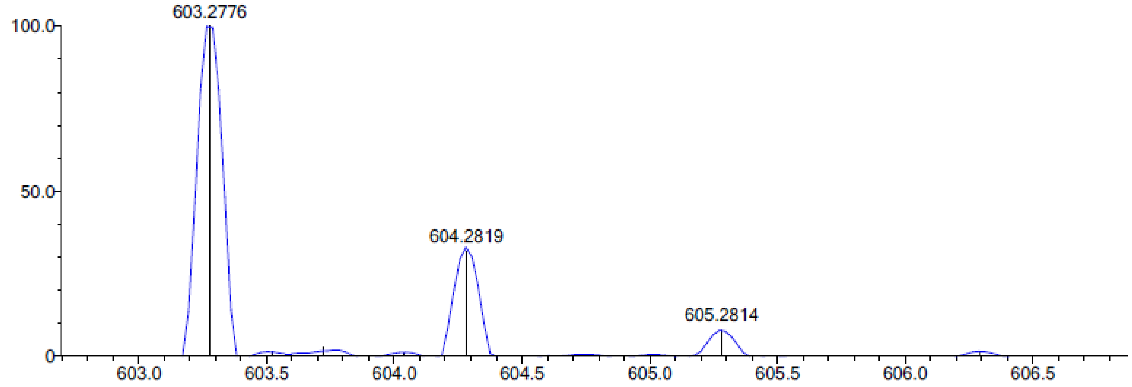

C30 H44 O11 [M+Na] + : Predicted region for 603.2776 m/z

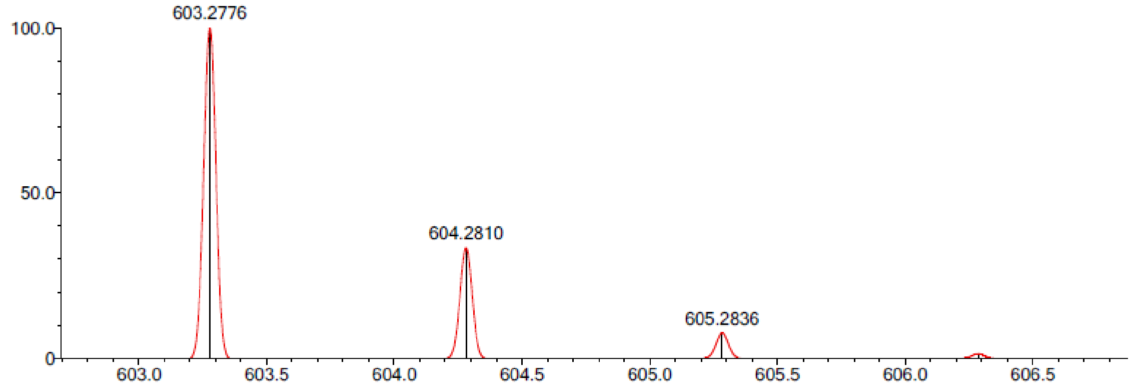

Rank	Score	Formula (M)	Ion	Meas. m/z	Pred. m/z	Df. (mDa)	Df. (ppm)	Iso	DBE
1	100.00	C30 H44 O11	[M+Na] +	603.2776	603.2776	0.0	0.00	100.00	9.0

Figure S13. ^{1}H NMR data of compound **3** recorded in CD_3OD at 500 MHz.

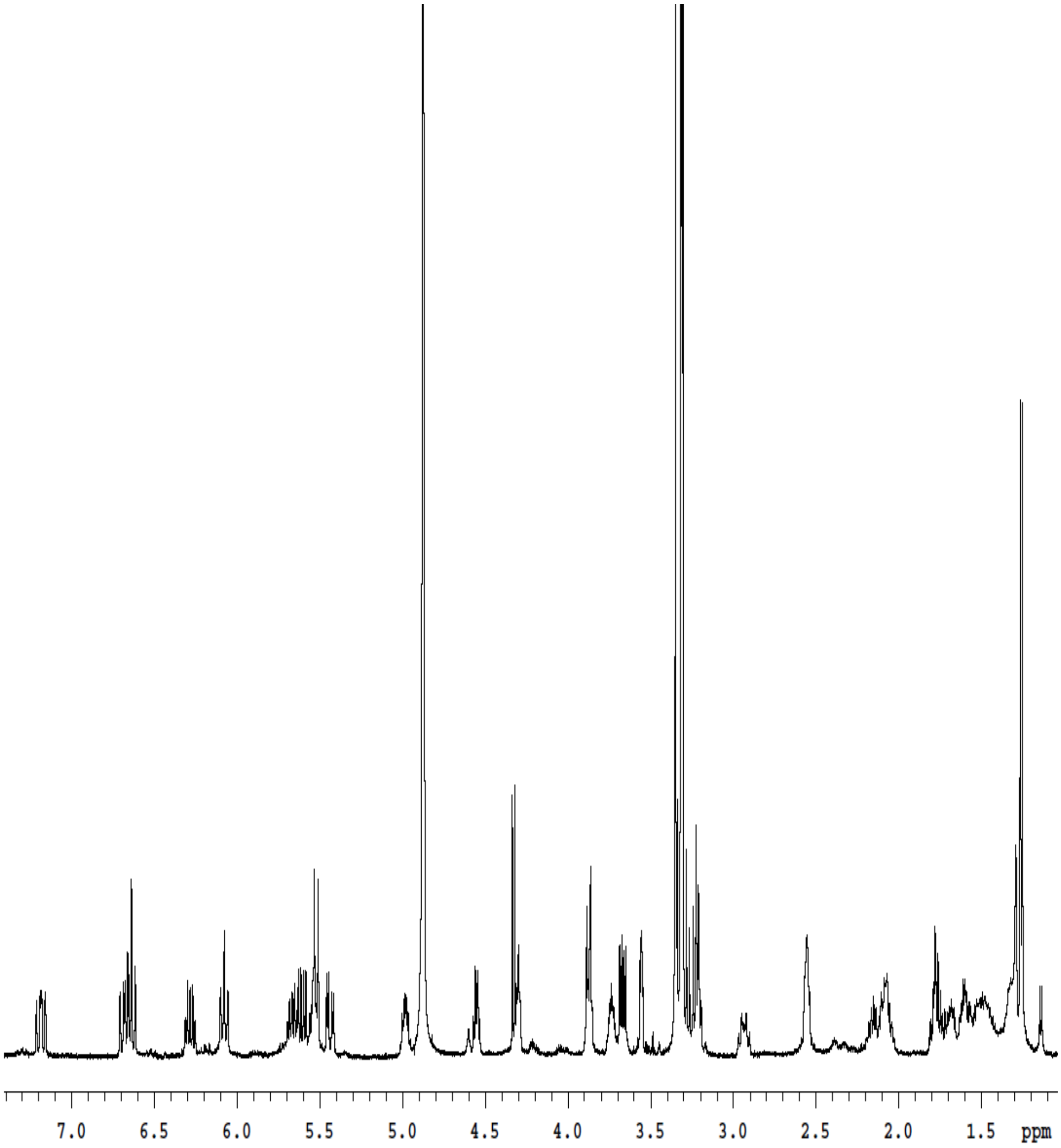

Figure S14. ^{13}C NMR data of compound **3** recorded in CD_3OD at 125 MHz.

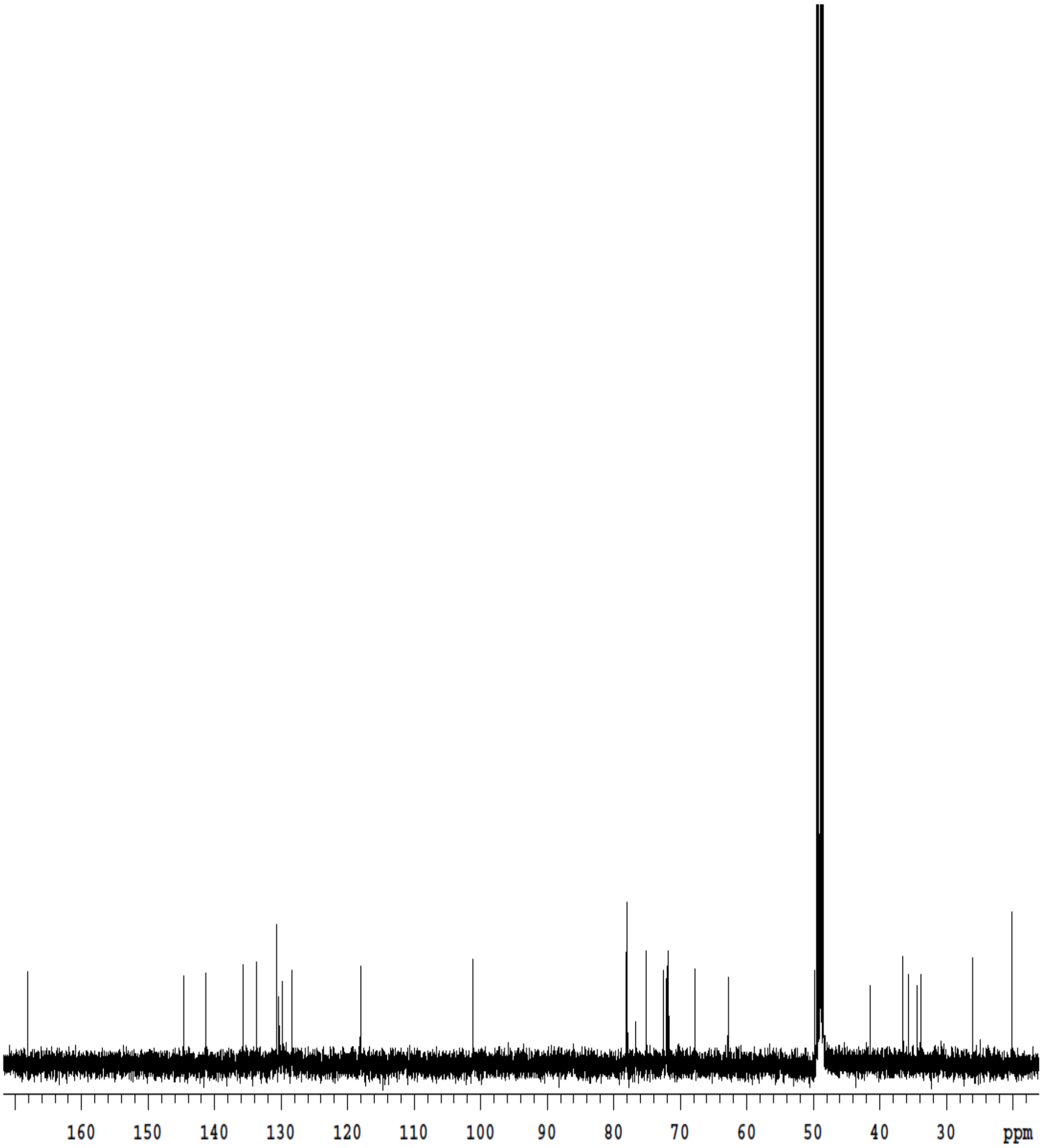

Figure S15. COSY spectrum of compound **3** in CD_3OD.

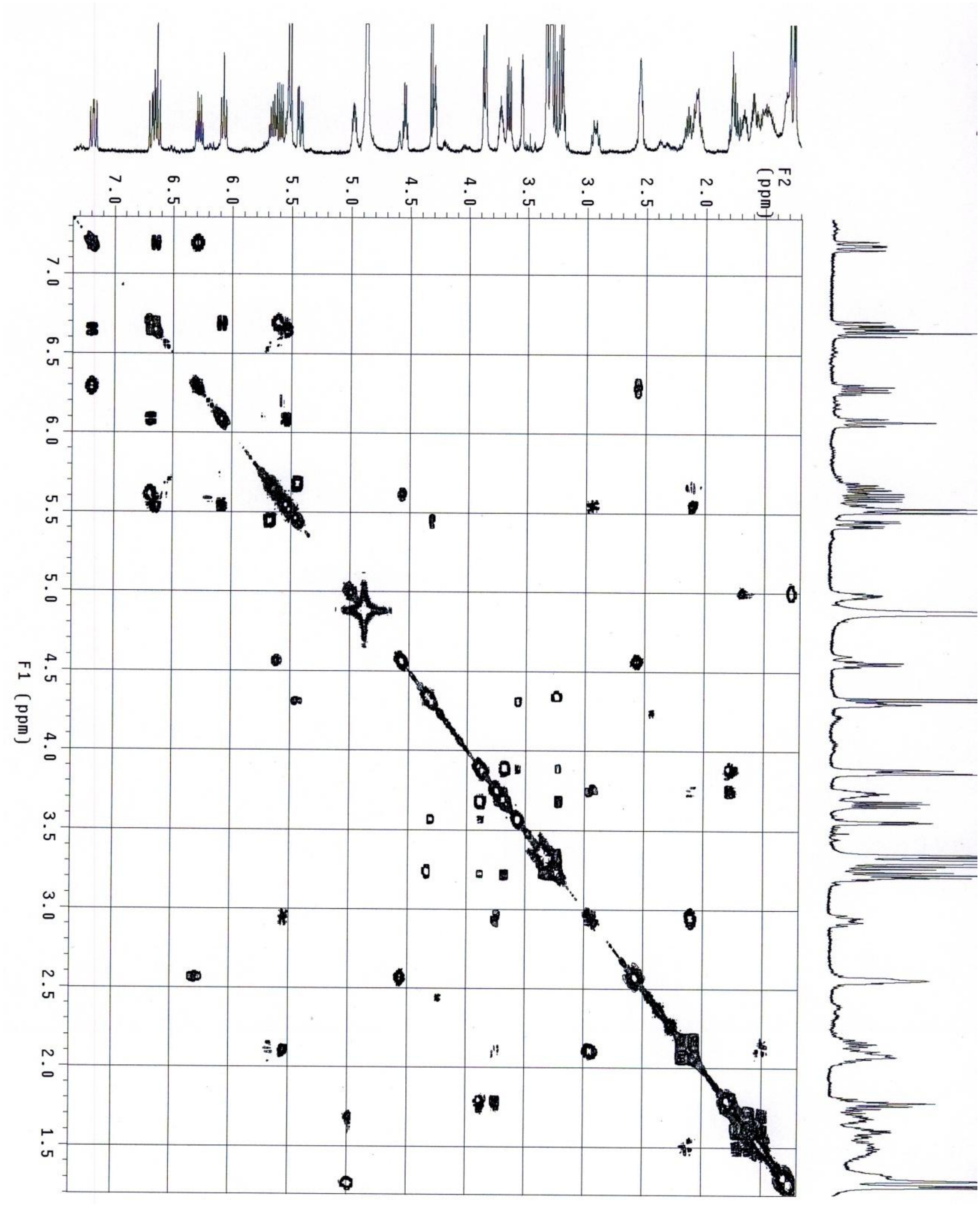

Figure S16. HSQC spectrum of compound **3** in CD_3OD.

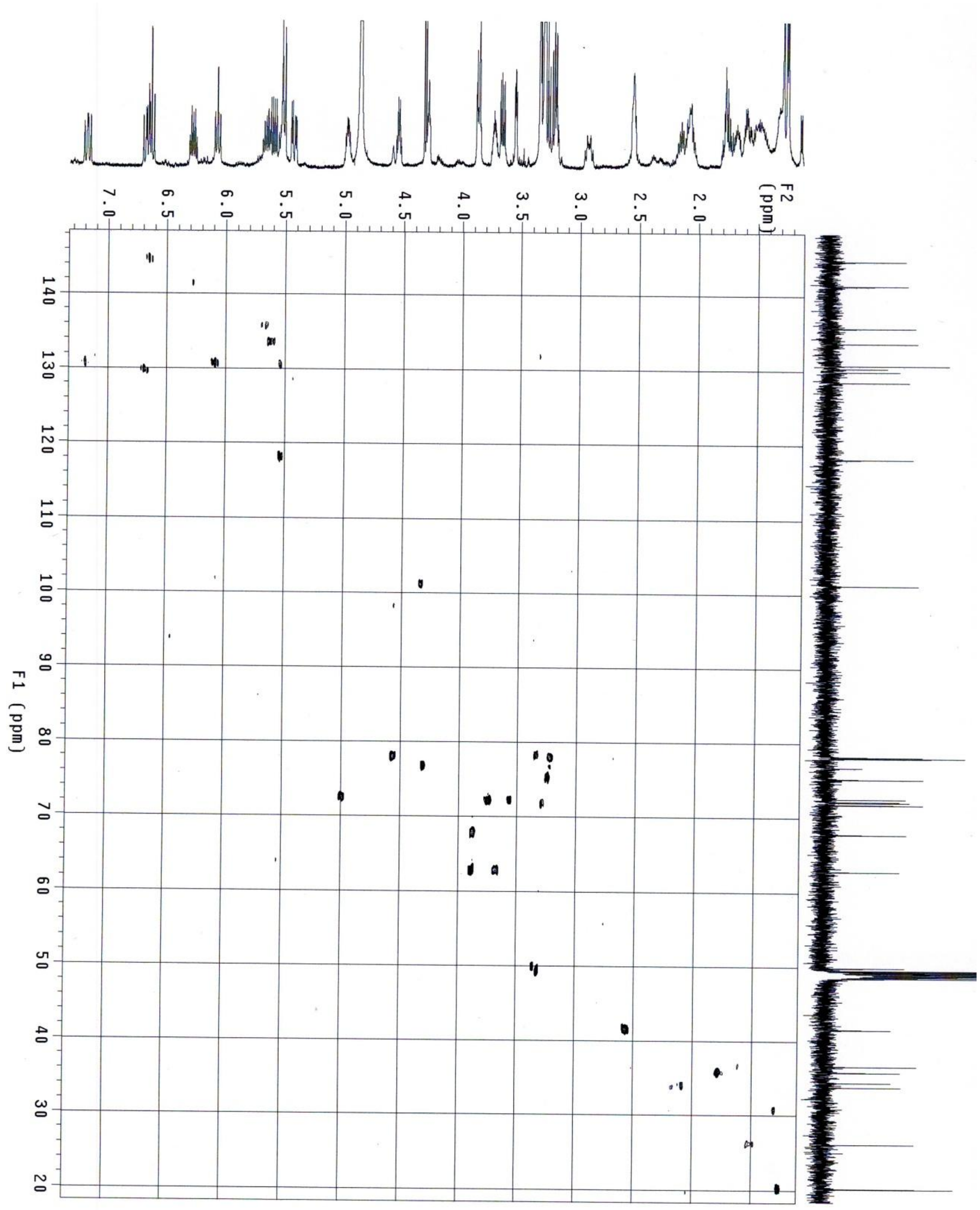

© 2014 by the authors; licensee MDPI, Basel, Switzerland. This article is an open access article distributed under the terms and conditions of the Creative Commons Attribution license (http://creativecommons.org/licenses/by/3.0/).
